# Supplementary material for: DNA damage during S-phase mediates the proliferation-quiescence decision in the subsequent G1 via p21 expression
Source: Nat Commun. 2017 Mar 20;8:14728. doi: 10.1038/ncomms14728 (PMC5364389; doi:10.1038/ncomms14728)
Supplement: Supplementary Information — Supplementary Figures, Supplementary Tables, and Supplementary Note [file ncomms14728-s1.pdf]

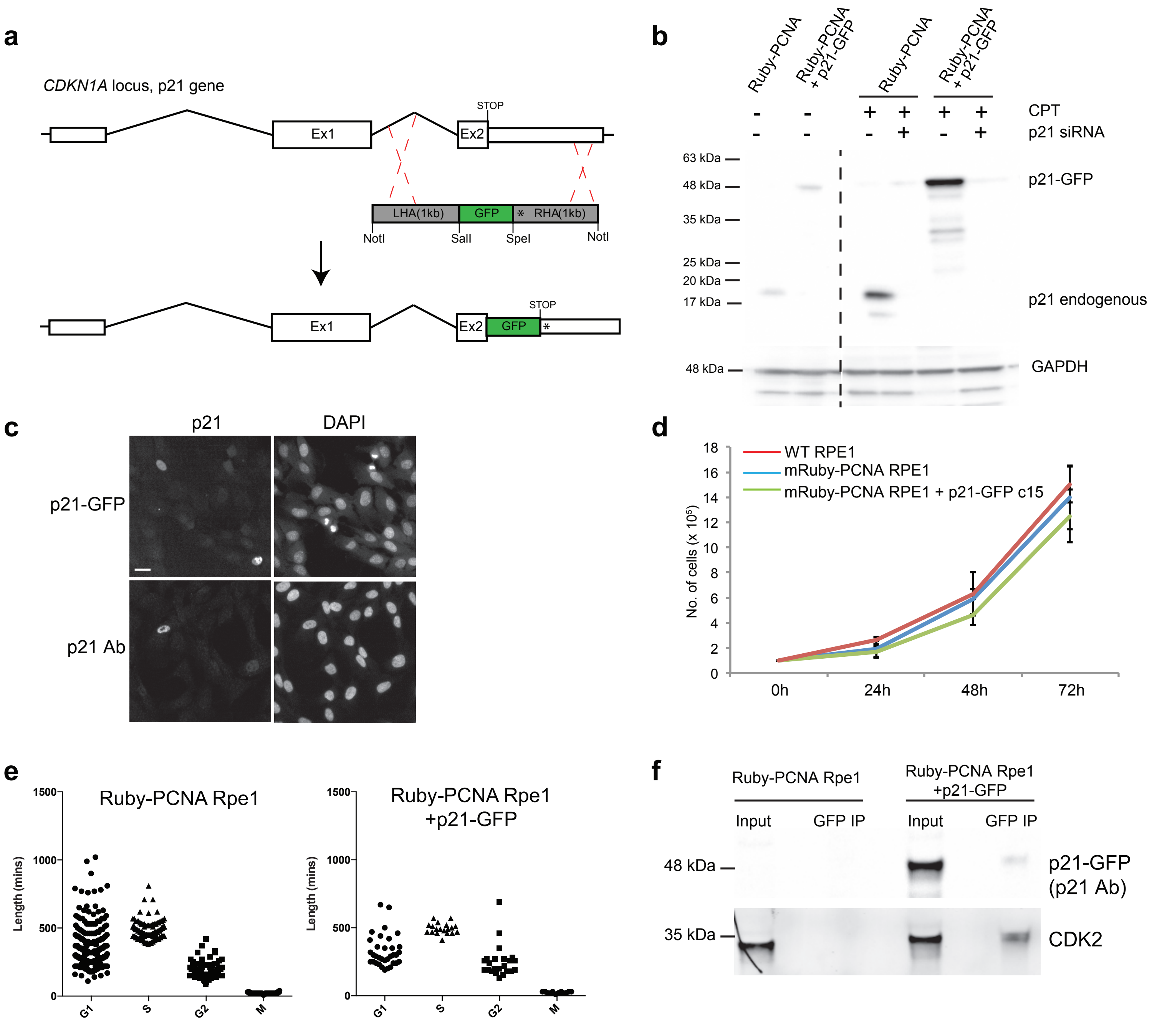

Supplementary Figure 1. a. Gene-targeting strategy for tagging p21 at the C-terminus with GFP. Asterisks represents mutation of PAM site. **b.** Western blot of p21-GFP expression indicating all p21 expressed in the hTert-RPE1 mRuby-PCNA p21-GFP cell line is labelled with GFP. Camptothecin (CPT) was added to induce DNA damage because p21 expression is low in unstimulated hTert-RPE1 cells (left-hand side of dashed line). p21 siRNA was used to confirm specificity of bands. Expected size of p21-GFP protein is 48 kDa. GAPDH was used as a loading control. **c.** p21 is nuclear in hTert-RPE1 cells. Top panels show p21-GFP localisation and bottom panels show hTert-RPE1 cells immunostained with a p21 specific antibody (p21 Ab). Scale bar is 10 µm. **d.** Tagging of p21 and PCNA does not affect growth rate of cells. Growth curves of wild-type (WT) hTert-RPE1 cells or hTert-RPE1 cells with mRuby-PCNA tag only or with mRuby-PCNA and p21-GFP. Mean +/- standard deviations are shown. n=3. **e.** C-terminal tagging of p21 does not perturb length of cell cycle phases or transition between phases. **f.** CDK2 coimmunoprecipitates with p21-GFP. Immunoprecipitation was performed against the GFP tag. Samples were run for western blotting with anti-p21 and CDK2 antibodies. Ruby-PCNA Rpe1 cells were used as a negative control.

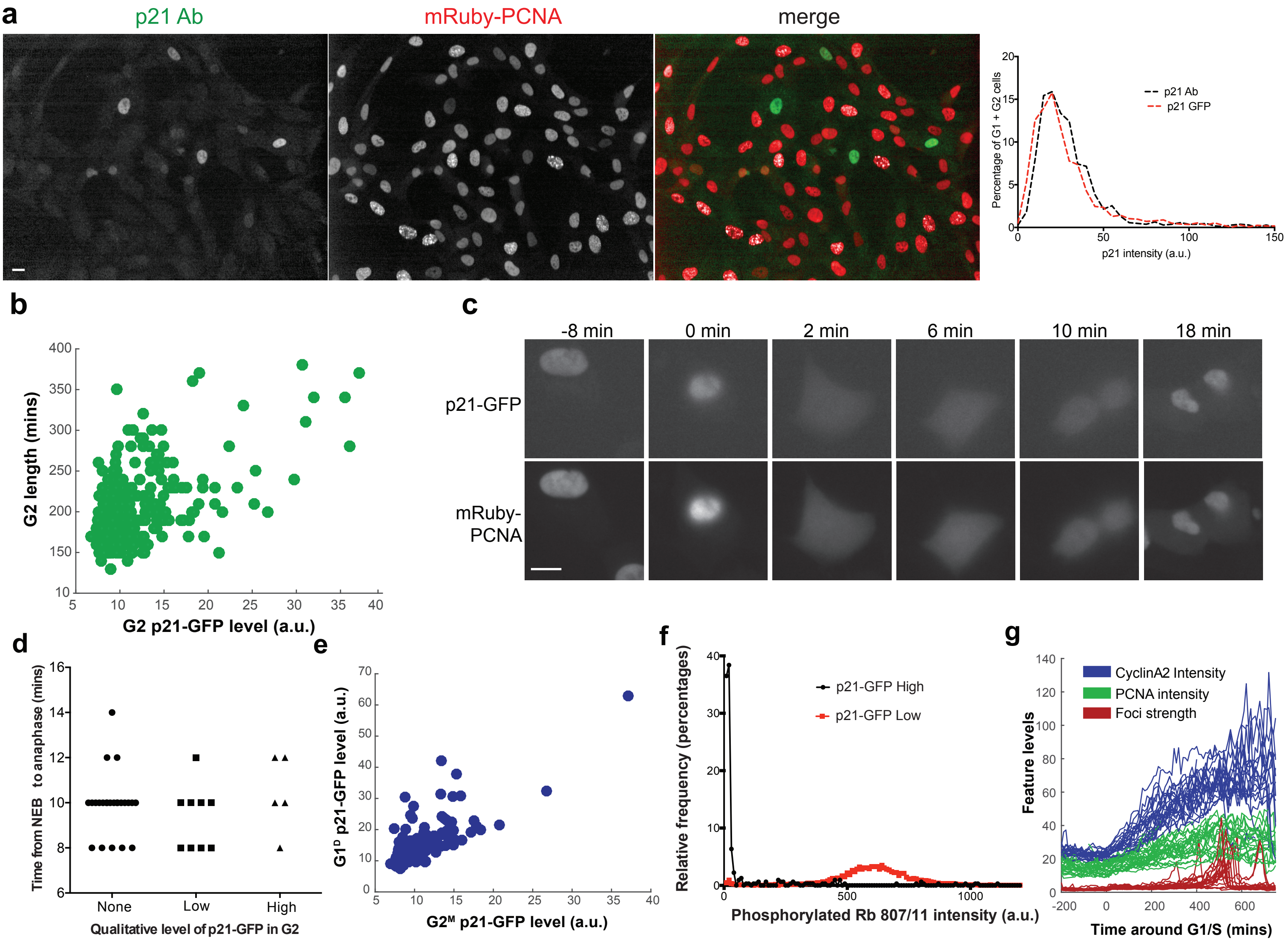

Supplementary Figure 2. a. p21 is absent in S-phase cells and expressed heterogeneously in G1 and G2 cells. Images show WT mRuby-PCNA cells immunostained with p21 antibody (Ab). p21 is absent from cells with PCNA foci (S-phase cells). Scale bar is 10 μm. Graph shows comparison of the quantification of the GFP signal in p21-GFP expressing cells (red curve) and immunostaining of p21 in WT mRuby-PCNA hTert-RPE1 cells (black curve). Endogenous p21 and p21-GFP have similar intensity distributions in G1 and G2 cells, indicating a similar level of heterogeneity. **b.** Correlation between maximum G2 p21-GFP intensity and G2 length (R = 0.52** (p<0.01)). **c.** p21-GFP is not degraded during mitosis and p21 expression does not perturb mitotic progression. Time is shown relative to nuclear envelope breakdown (t=0 min) when both p21 and PCNA fluorescent signals become dispersed in the cytoplasm but are not degraded. Scale bar is 10 μm. **d.** Graph shows the qualitatively assessed level of p21-GFP expressed in G2 and the effect on the length of mitosis. **e.** Correlation between p21-GFP intensities in $G2^M$ and $G1^D$ (one of the two daughter cells was randomly selected; R = 0.75** (p<0.01)). **f.** Fixed cells expressing high p21-GFP (>150 a.u.) have hypophosphorylated Rb, indicative of arrest before the Restriction Point (RP). **g.** CyclinA2-mVenus levels (Blue) increase following G1/S transition, as we define by mRuby-PCNA increase (Green) and a small jump in the PCNA foci strength measure (Red).

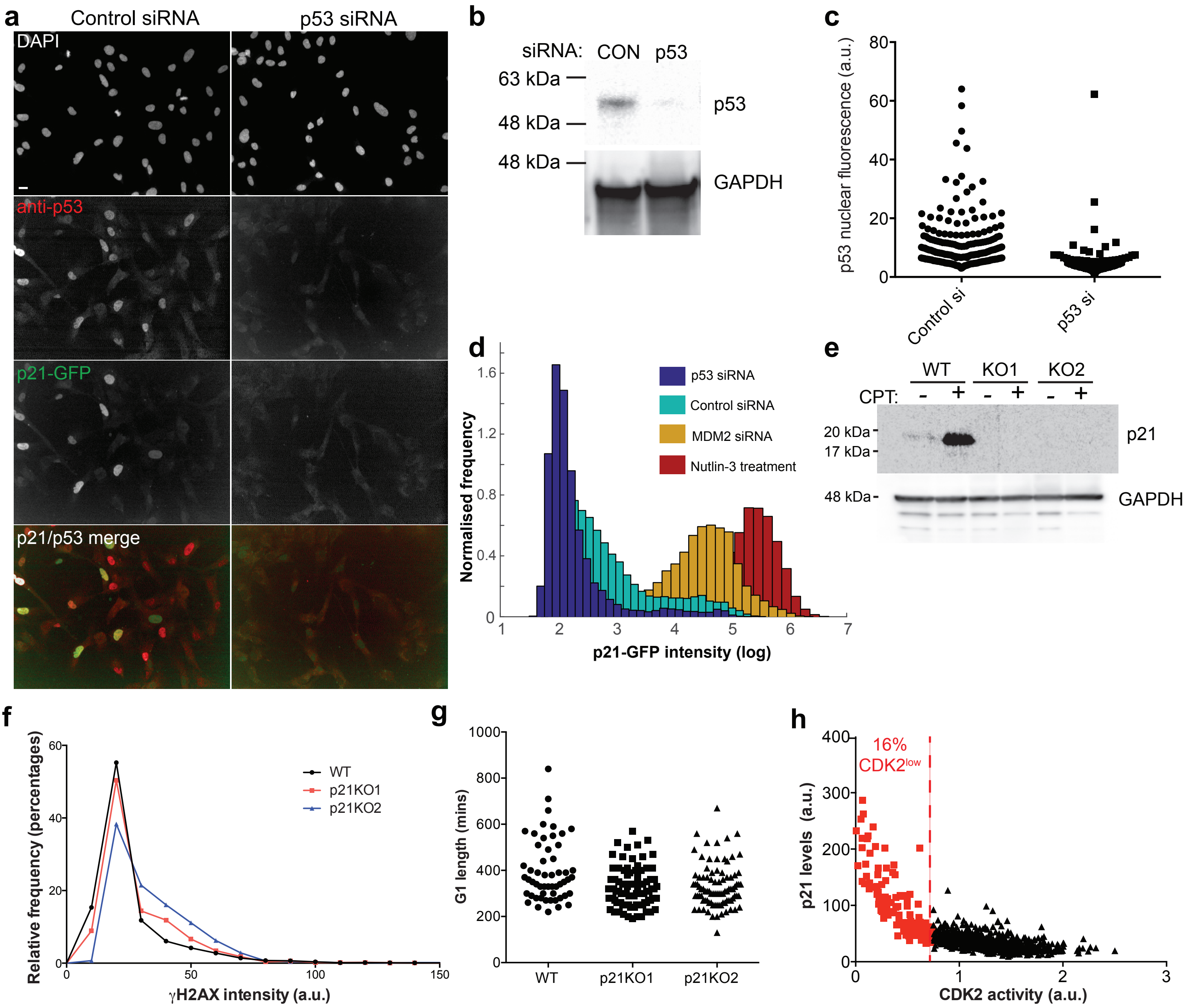

Supplementary Figure 3. a. Images showing p21 expression after p53 depletion. Representative fields of view are shown for hTert-RPE1 mRuby-PCNA p21-GFP cells treated with control (left panels) or p53 siRNA (right panels) and fixed 48 hr later. p21 is green, p53 is red in merged images. Scale bar is 10 μm. **b.** Western blot shows p53 depletion after 48 hr siRNA. GAPDH is used as a loading control. **c.** Quantification of nuclear p53 levels in cells treated with either control or p53 siRNA and immunostained for p53. Single cell data for 238 control siRNA and 218 p53 siRNA treated cells is shown. **d.** p21-GFP intensity under different treatment conditions. Compared to cells treated with control siRNA, p53 siRNA leads to a reduction in p21-GFP levels, whereas treatment with Nutlin-3 leads to an increase in p21-GFP levels. **e.** Western blot showing absence of full-length p21 protein in p21KO cell lines. To induce p21 expression, 1 μM CPT was added for 24 hr before cell lysis. Two independent nickase constructs were used to generate clonal KO lines (see Supplementary Information; (Chiang et al. 2016)). GAPDH was used as a loading control. **f.** p21KO cells have higher basal levels of DNA damage, as shown by increased γH2AX levels, measured by immunostaining. n=3505, WT; n=2743, p21KO1; n=3490 p21KO2. **g.** Distribution of G1 lengths for wild-type (WT) versus p21KO1 and p21KO2 clones. **h.** Scatter plot shows p21 levels versus CDK2 activity in G1 in untreated hTert-RPE1 mRuby-PCNA CDK2 sensor expressing cells, fixed and immunostained with anti-p21. CDK2low (< 0.75 a.u.) cells represent 16% of the population (red dots), which correlates with the percentage of cells that we observe undergoing G1 arrest in unperturbed conditions in live imaging experiments (13-21%).

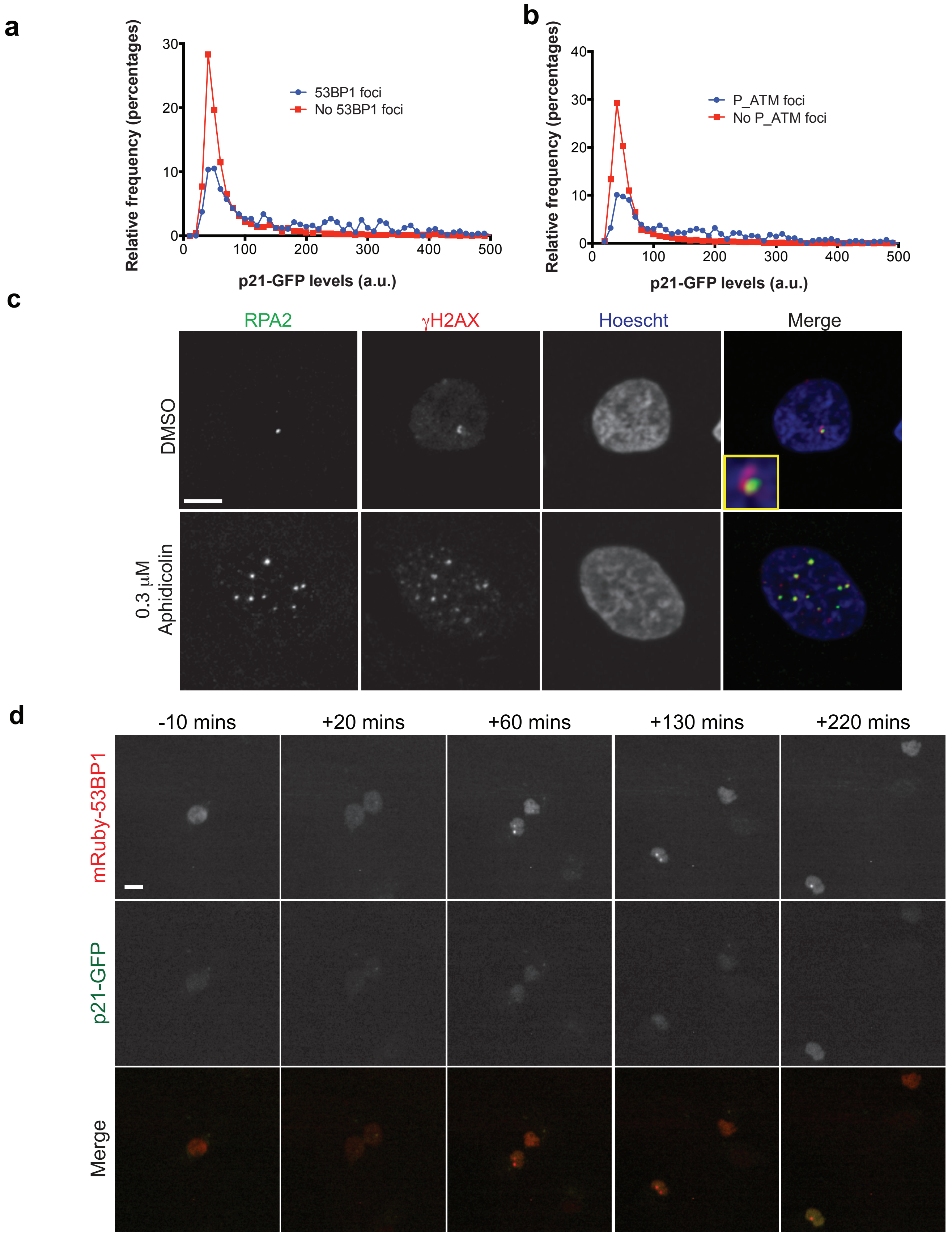

Supplementary Figure 4. a. Nuclear p21-GFP levels in G1 and G2 cells with at least one nuclear 53BP1 focus versus cells with no foci. (n=561, with foci; n=2388, no foci). **b.** Nuclear p21-GFP levels in G1 and G2 cells with at least one nuclear PS1981_ATM focus versus cells with no foci. (n=564, with foci; n=2446, no foci). **c.** Images of hTert-RPE1 cells immunostained for γH2AX and the ssDNA marker RPA2. Cells treated with DMSO show evidence of ssDNA breaks in G2 (cell cycle phase determined by the presence of condensed chromatin), since RPA2 colocalises with γH2AX in these cells. After 24 hr treatment with 0.3 μM aphidicolin, the number of ssDNA foci in G2 cells increases. RPA2 is in green, γH2AX is in red, DNA is in blue in merged images. Scale bar is 10 μm. **d.** Stills from Movie S4 showing co-segregation of p21-GFP with a mRuby-53BP1 in G1. At t=60 min, the lower cell displays two 53BP1 foci and this cell expresses high levels of p21-GFP. Time refers to nuclear envelope breakdown (0 min). mRuby-53BP1 is red, p21-GFP is green in merged image. Scale bar is 10 μm.

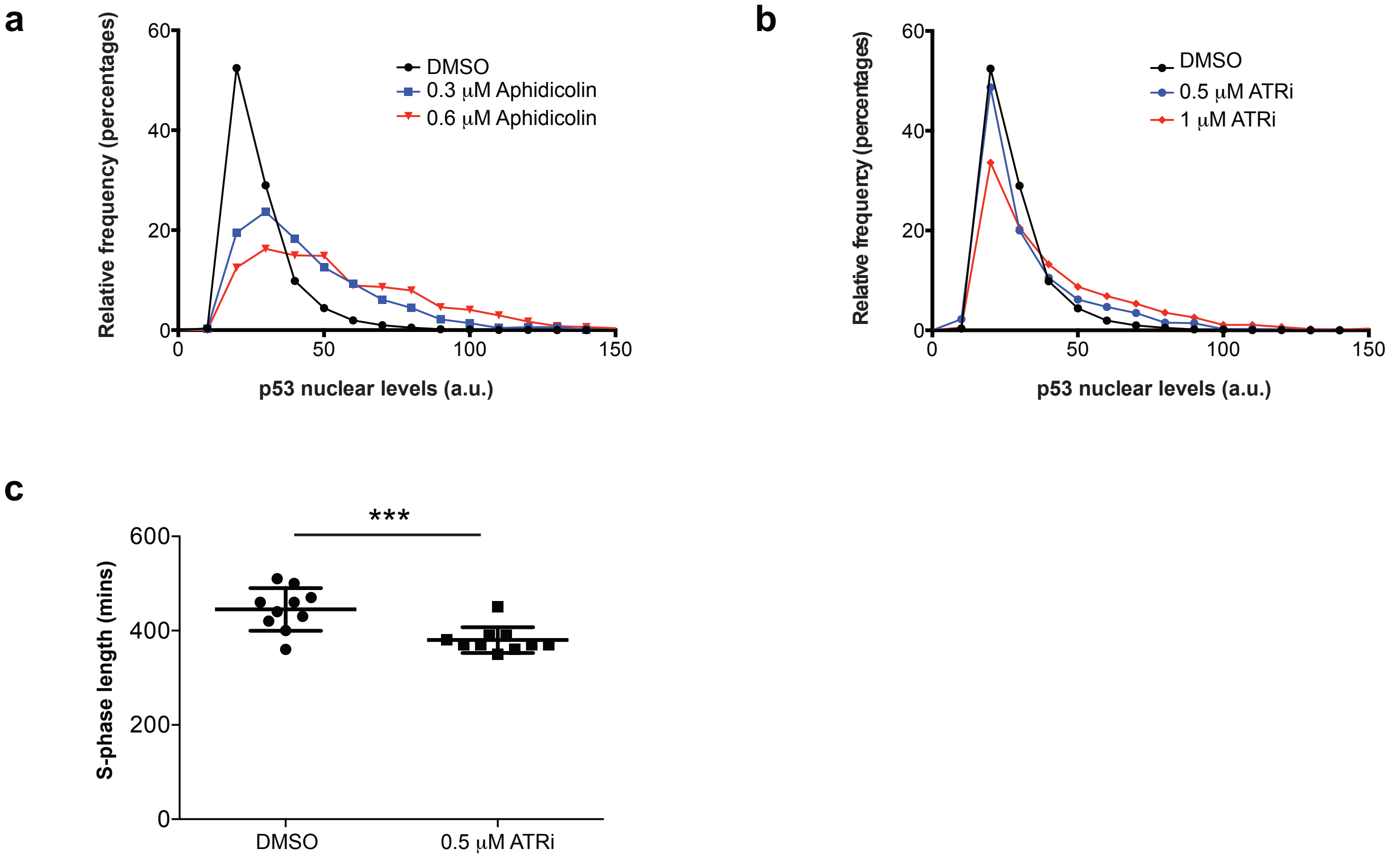

Supplementary Figure 5. a. p53 expression levels after 24 hr treatment of hTert-RPE1 cells with aphidicolin. (n=2195, DMSO; n=1149, 0.3 μM Aph; n=983, 0.6 μM Aph). **b.** p53 expression levels after 24 hr treatment of hTert-RPE1 cells with ATR inhibitor (AZD6738). (n=2195, DMSO; n=2381, 0.5 μM ATRi; n=2224, 1 μM ATRi). **c.** S-phase length in DMSO- versus ATRi-treated cells. Unpaired student's t-test, $p<0.001$.

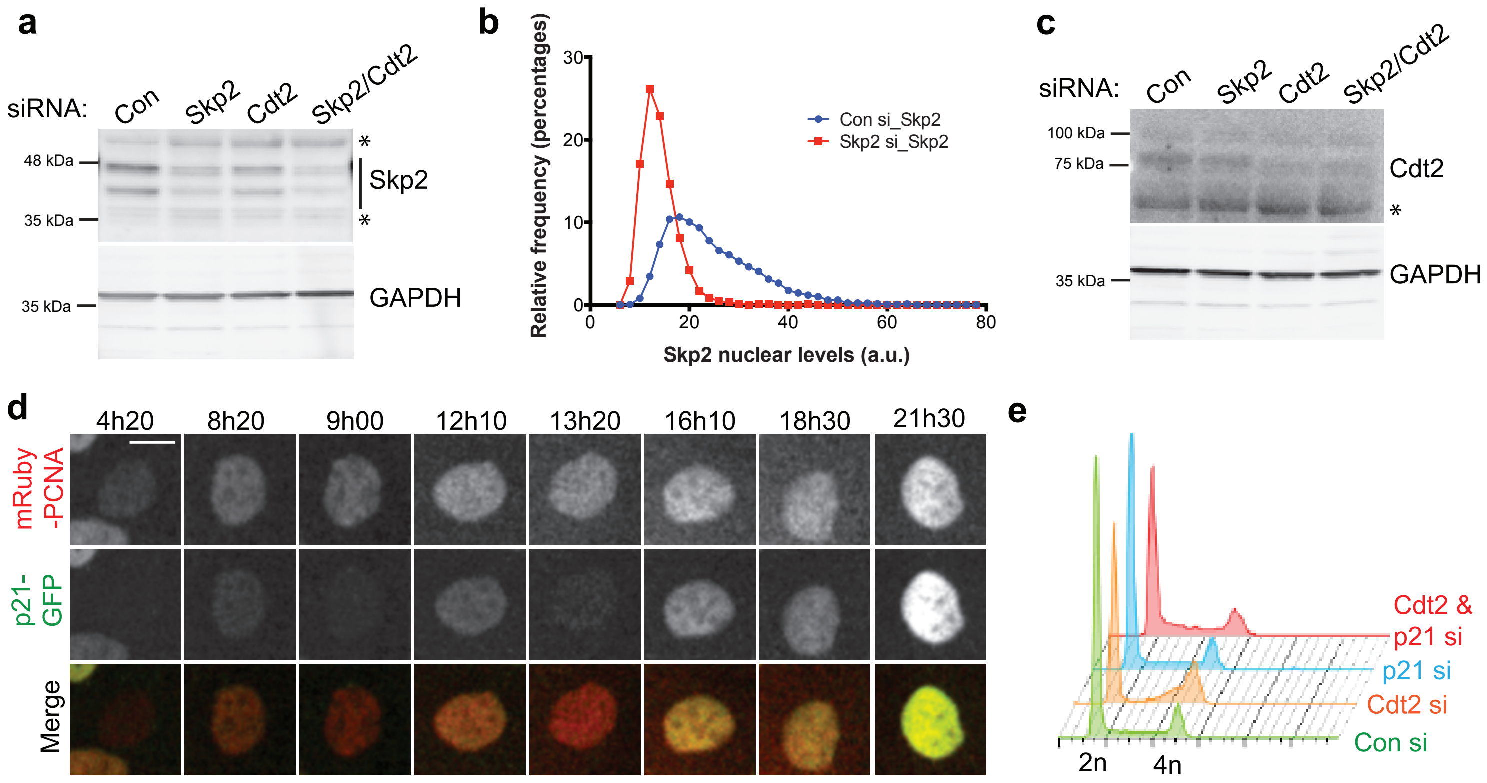

Supplementary Figure 6. a. Western blot showing depletion of Skp2 protein by Skp2 siRNA, both in cells depleted of Skp2 only and cells co-depleted of Skp2 and Cdt2. GAPDH is used as a loading control. Asterisks mark non-specific bands. **b.** Quantification of Skp2 levels in individual nuclei from immunostaining of Skp2-depleted cells, 48 hr after siRNA. (n=7365, Con si; n=5789, Skp2 si). **c.** Western blot showing depletion of Cdt2 protein by Cdt2 siRNA, both in cells depleted of Cdt2 only and in cells co-depleted of Cdt2 and Skp2. GAPDH is used as a loading control. Asterisk marks non-specific band. **d.** Stills taken from Movie S5 showing oscillations in p21-GFP levels in Cdt2-depleted cells. Cell enters S-phase at 4h20. mRuby-PCNA is in red, p21-GFP is in green in merged image. Scale bar is 10 μm. **e.** FACS plots showing an accumulation of cells in late S and G2 in Cdt2-depleted cells (orange curve). This phenotype was rescued by Cdt2 and p21 co-depletion (red curve).

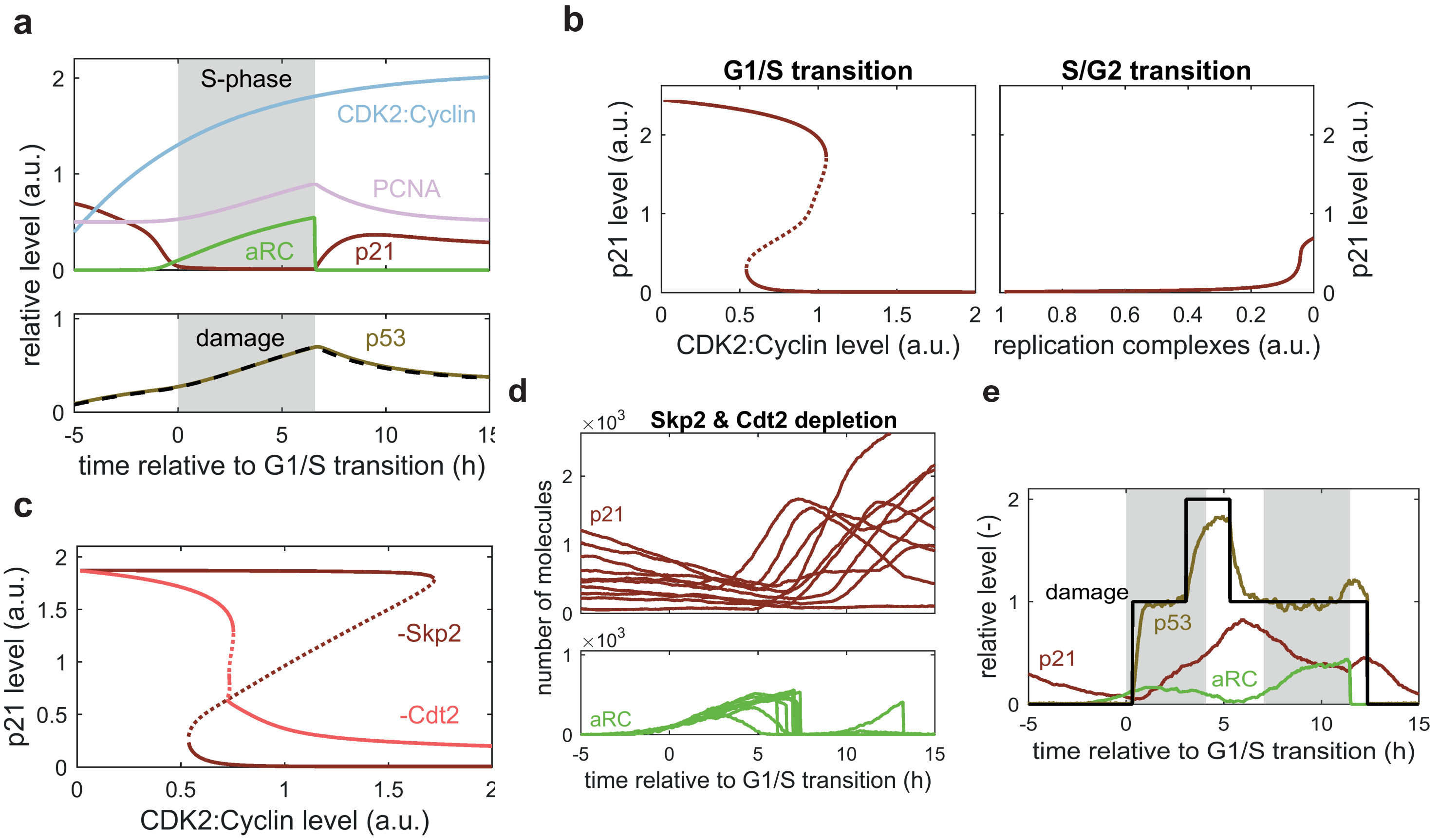

Supplementary Figure 7. a. Deterministic simulation of the mathematical model. Time courses of model components are shown relative to the G1/S transition. Grey shaded regions indicate Sphase. **b.** Stable (solid) and unstable (dashed) steady states of p21 during the G1/S transition (left panel) and the S/G2 transition (right panel) in the presence of low levels of DNA damage. **c.** Stable (solid) and unstable (dashed) steady states of p21 with respect to CDK2:Cyclin in Skp2 and Cdt2-depleted cells. **d.** Stochastic simulation of p21 (upper panel) and active replication complexes (aRC; lower panel) in cells depleted for Skp2 and Cdt2. Ten representative cells are shown. **e.** Premature S-phase exit upon p21 expression. Example of a simulated Cdt2-depleted cell that suffered DNA damage during S-phase causing p21 re-accumulation and inhibition of DNA synthesis.

Supplementary Note 1. Genotyping of p21KO clones 1A and 2A.

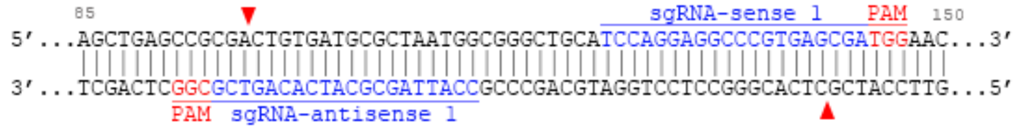

Clone	Sequence (5'- 3')	Indel
WT	AGCTGAGCCGCGACTGTGATGCGCTAATGGCGGGCTGCATCCAGGAGGCCCGTGAGCGATGGAAC	
1A	AGCTGAGCCGCGACTG--------------------------------CCCGTGAGCGATGGAAC	-32bp

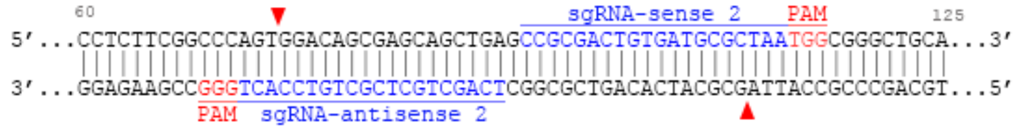

Clone	Sequence (5'- 3')	Indel
WT	CCTCTTCGGCCCAGTGGACAGCGAGCAGCTGAGCCGCGACTGTGATGCGCTAATGGCGGGCTGCA	
2A	CCTCTTCGGCCCAGTGGACAGCGAGCAGCTGA▲[1]GCCGCGACTGTGATGCGCTAATGGCGGGCTGCA	+48bp
	CCTCTTCGGC▲[2]CCAGTGGA▲[3]CAGCG▲[4]AGCAGCTGAGCCGCGACTGTGATGCGCTAATGGCGGGCTGCA	+68bp

[1] CTGAGGCCGCCCGGGCACATCAGCCGCGACTGTGATGCGCGCATCA
[2] GAAGTCACCCT
[3] TGTCTCGGTGACAAAGTCGAAGTTCCATCGCTCACGGGCCTCCTGGAT
[4] CCGCCATTA

Clone	Protein Sequence	Size
WT	MSEPAGDVRQNPCGSKACRRLFGPVDSEQLSRDCDALMAGCIQEARERWNFD...	164AA
1A	MSEPAGDVRQNPCGSKACRRLFGPVDSEQLSRDCP*	35AA
2A	MSEPAGDVRQNPCGSKACRRLFGPVDSEQLTEAARAHQPRL*	41AA
	MSEPAGDVRQNPCGSKACRRLFGEVTLQWCLGDKVEVPSLTGLLDAARH*	49AA

Supplementary Table 1. Summary of R- and P-values for Pearson's correlations stated in the main text.

Correlation	Mean p21 level		Maximum p21 level		log(time) vs log(max) p21	
	R	P	R	P	R	P
G1 length to p21 level	0.5939	4.1231e-19	0.6163	7.6580e-21	0.5616	5.4876e-17
G2 length to p21 level	0.4528	1.3023e-15	0.5146	2.1324e-20	0.4719	1.6389e-14
S-phase length to p21 level	-0.0741	0.3146	0.0720	0.3285	0.0306	0.6785
G2 Mother p21 level to G1 Daughter p21 level	0.7012	1.2832e-26	0.7482	6.2568e-32	0.7166	3.0996e-28
G2 Mother length to G1 Daughter length	R = 0.0707<br>P = 0.3580					
G1 Mother p21 levels to G1 Daughter p21 levels	0.5272	0.0023	0.5075	0.0036	0.4247	0.0173
G1 Mother length to G1 Daughter length	R = 0.1258<br>P = 0.5002					
G1 Daughter p21 levels to G2 Mother + G1 Daughter length	0.6687	1.6250e-23	0.6834	7.2175e-25	0.6445	1.8888e-21
G1 Daughter p21 levels to G2 Daughter p21 levels	0.3308	2.7011e-04	0.3063	7.8206e-04		
G1 Daughter length to G2 Daughter length	R = 0.1607<br>P = 0.0835					

Supplementary Table 2. R-values for Pearson's correlations between phase length and maximum p21 value for different experimental repeats.

	Length of $G1^D$ vs p21 max	Length of $G2^M$+$G1^D$ vs p21 max
Repeat 1	0.3980	0.5993
Repeat 2	0.7671	0.8144
Repeat 3	0.7444	0.7951
Repeat 4	0.6991	0.7254

Supplementary Table 3.

**Parameters of the mathematical model.**			
**Parameter**	**Description**	**Value**	**Unit**
$Skp2$	relative Skp2 level	1 (0.01*)	AU
$Cdt2$	relative Cdt2 level	1 (0.01*)	AU
j_{Cy}	CDK2 threshold for RC priming	1.8	AU
j_{Dam}	DNA damage threshold for repair	0.5	AU
j_{p53}	inhibition constant of p53 degradation	0.01	AU
$k_{\mathrm{CyP21}}^{\mathrm{As}}$	association of CDK2:Cyclin and p21	1	1/(AU·min)
$k_{\mathrm{PcP21}}^{\mathrm{As}}$	association of PCNA and p21	100	1/(AU·min)
$k_{\mathrm{RcPc}}^{\mathrm{As}}$	association of primed RCs and PCNA	0.01	1/(AU·min)
$k_{\mathrm{Cy}}^{\mathrm{De}}$	constitutive cyclin degradation	0.002	1/min
$k_{\mathrm{Cy,Cy}}^{\mathrm{De}}$	CDK2:Cyclin-mediated cyclin degradation	0.0002	1/(AU·min)
$k_{\mathrm{mRNA}}^{\mathrm{De}}$	mRNA degradation	0.02	1/min
$k_{\mathrm{p21}}^{\mathrm{De}}$	constitutive p21 degradation	0.0025	1/min
$k_{\mathrm{p21,Cy}}^{\mathrm{De}}$	CDK2:Cyclin-mediated p21 degradation	0.007	1/(AU·min)
$k_{\mathrm{p21,RCa}}^{\mathrm{De}}$	RC_a-mediated p21 degradation	1	1/(AU·min)
$k_{\mathrm{p53}}^{\mathrm{De}}$	DNA damage-dependent p53 degradation	0.05	AU/min
$k_{\mathrm{Rc}}^{\mathrm{Dp}}$	dephosphorylation of primed RCs	0.01	1/min
$k_{\mathrm{CyP21}}^{\mathrm{Ds}}$	dissociation of CDK2:Cyclin:p21 complexes	0.05	1/min
$k_{\mathrm{PcP21}}^{\mathrm{Ds}}$	dissociation of PCNA:p21 complexes	0.01	1/min
$k_{\mathrm{RcPc}}^{\mathrm{Ds}}$	dissociation of RC_p:PCNA complexes	0.001	1/min
$k_{\mathrm{Pc}}^{\mathrm{Ex}}$	PCNA export from the nucleus	0.006	1/min
$k_{\mathrm{Dam}}^{\mathrm{Ge}}$	replication-independent DNA damage	0.001	AU/min
$k_{\mathrm{Dam,RCa}}^{\mathrm{Ge}}$	replication-dependent DNA damage	0.005	1/min
$k_{\mathrm{Pc}}^{\mathrm{Im}}$	PCNA import into the nucleus	0.003	AU/min
$k_{\mathrm{Rc}}^{\mathrm{Ph}}$	CDK2:Cyclin-mediated priming of RCs	0.1	1/min
$k_{\mathrm{Dam}}^{\mathrm{Re}}$	p53-independent DNA damage repair	0.001	1/min
$k_{\mathrm{Dam,p53}}^{\mathrm{Re}}$	p53-dependent DNA damage repair	0.005	1/min
$k_{\mathrm{Cy}}^{\mathrm{Sy}}$	cyclin synthesis	0.005	1/min
$k_{\mathrm{Dna}}^{\mathrm{Sy}}$	DNA synthesis by active RCs	0.007	1/min
$k_{\mathrm{mRNA}}^{\mathrm{Sy}}$	constitutive mRNA synthesis	0.02	AU/min
$k_{\mathrm{mRNA,p53}}^{\mathrm{Sy}}$	p53-dependent synthesis of p21 mRNAs	0.08	1/min
$k_{\mathrm{p21}}^{\mathrm{Sy}}$	constitutive p21 synthesis	0.0018	1/min
$k_{\mathrm{p53}}^{\mathrm{Sy}}$	constitutive p53 synthesis	0.05	1/min
n	Hill coefficient for priming of RCs	6	-

*in simulations of depletion experiments

Supplementary Table 4.

Non-zero initial conditions of the mathematical model.			
**Variable**	**Description**	**Value**	**Unit**
$mRNA_{\mathrm{Cy}}$	mRNAs encoding for cyclins	1	AU
$mRNA_{\mathrm{p21}}$	mRNAs encoding for p21	1	AU
$mRNA_{\mathrm{p53}}$	mRNAs encoding for p53	1	AU
$p21_{\mathrm{t}}$	total level of p21	0.72	AU
$PCNA_{\mathrm{a}}$	level of free PCNA in the nucleus	0.5	AU
RC	level of pre-replication complexes	1	AU
